# Participatory Design in Suicide Prevention: A Qualitative Study of International Students' Experiences of Adapting the LivingWorks safeTALK Programme

**DOI:** 10.1111/hex.14164

**Published:** 2024-08-06

**Authors:** Samuel McKay, Christina Ng, Bridget Kenny, Rafi Armanto, Michelle Lamblin, Jo Robinson

**Affiliations:** ^1^ Orygen Parkville Victoria Australia; ^2^ Centre for Youth Mental Health, The University of Melbourne Parkville Victoria Australia

**Keywords:** co‐consultation, cultural adaptation, culturally responsive, foreign students, programme adaptation, suicide alertness

## Abstract

**Background:**

Current suicide prevention approaches are not adapted to international student needs, and participatory design is a method that may facilitate the development or adaptation of appropriate programmes for this group.

**Methods:**

This qualitative study investigated the experiences of international university students studying in Australia who participated in a co‐consultation process to adapt the LivingWorks safeTALK suicide prevention programme. Eight international students from the co‐consultation workshop completed semi‐structured interviews about their workshop experience. The data were analysed using reflexive thematic analysis.

**Results:**

The findings showed that participants found the co‐consultation process empowering and engaging. They also reported that the experience promoted mutual learning and challenged simplistic views of suicide. No students reported experiencing distress. Suggestions for improving participatory design for international students focussed on enhancing participant interaction, supporting quiet voices to be heard and ensuring understanding of mental health and suicide through shared language.

**Conclusions:**

This study underscores the value of participatory design in suicide prevention, emphasising its potential to empower international students and facilitate culturally sensitive programme adaptations.

**Patient or Public Contribution:**

International students were involved in the co‐consultation process to redevelop the training content and provided a series of recommendations for improving such processes for international students in the future. The two researchers who conducted the interviews and data analysis were former international students.

## 1 Introduction

International students who come to another country to complete tertiary education are a population who report high rates of suicidal thoughts (12‐month prevalence: 5.6%–9.8%) and behaviours (12‐month prevalence: 1.2%–2.2% [1]). Yet, no suicide prevention programmes specifically designed for international students are currently available [2]. To address this gap, new prevention initiatives are needed. Participatory design methodologies, such as co‐design and co‐consultation, have shown promise in enhancing the acceptability, effectiveness and uptake of interventions with international students and young people [3, 4, 5]. However, there is a notable paucity of research evaluating these participatory approaches in suicide prevention, particularly for international students [2, 6]. This gap leaves unanswered questions about the appropriateness, safety and feasibility of participatory design approaches for suicide prevention with international students. The current qualitative study seeks to bridge this gap by exploring the experiences of tertiary‐level international students in Australia who were involved in a co‐consultation process to refine the LivingWorks safeTALK programme to better cater to their needs.

Suicide ranks as a primary cause of mortality among young people [7], with individuals in the tertiary education age group (18–25) being especially vulnerable [8]. Research indicates that international students, while reporting suicidal thoughts at rates similar to their domestic counterparts, exhibit a higher propensity for suicide attempts [1, 9]. Compounding this issue is the observed tendency of international students to seek help less frequently than other student groups [10], a pattern that was found to be consistent in coronial investigations into several international student suicide deaths in Australia [11, 12, 13, 14, 15, 16]. This underscores the need for tailored mental health interventions and support systems within academic environments for this group.

While no evidence‐based interventions specifically designed for international students are available, several prevention programmes relevant to tertiary education settings exist [17, 18]. These programmes can be categorised using the indicated, selective and universal prevention framework [17]. For indicated prevention, targeted interventions like dialectical behaviour therapy (DBT), designed for individuals already exhibiting suicidal thoughts and behaviours, have shown promise. One study showed that DBT outperformed treatment as usual in reducing suicidal thoughts and behaviours at a college counselling centre [19]. Selective prevention strategies, such as targeted risk screening and referral programmes, aim to identify and support individuals at elevated risk. These programmes have been found to have only modest effects [18]. Universal prevention approaches, particularly gatekeeper training programmes targeting the broader population, have the largest available evidence base. A meta‐analysis showed that these programmes improve participants' suicide‐related knowledge and feelings of self‐efficacy to support others experiencing suicidal thoughts or behaviours [18].

Gatekeeper training programmes are designed to equip members of a community with the skills to identify those at risk of suicide, understand the factors that contribute to their risk and connect them with appropriate support services [20]. Gatekeeper training has been adapted for numerous education settings and cultural groups [21, 22, 23]. However, participatory design is uncommon when adapting these programmes, and gatekeeper training has not been adapted for international students [2]. This is a significant gap not only because of the vulnerability within the international student community but also because international students comprise a broad range of nationalities, cultures and beliefs and have diverse needs [24]. The diversity within the international student community represents a particular challenge when developing prevention programmes that are inclusive and adapted to cultural groups [2]. Participatory research approaches may overcome this issue by identifying the shared experiences across groups that universal prevention programmes such as gatekeeper training can target [25].

Adopting a participatory research approach, which actively involves the target audience in the decision‐making process during the adaptation of initiatives, is widely recommended and increasingly implemented in suicide prevention [26, 27]. This methodology ensures that the perspectives and needs of those directly impacted by the initiative are considered, which can lead to more effective and tailored outcomes [28]. Although the advantages of participatory research approaches are widely recognised, there are challenges in effectively engaging individuals from culturally and linguistically diverse (CALD) groups in these processes [3]. Key questions persist regarding the most effective methods to include these groups to ensure their experiences and perspectives are genuinely understood and integrated. Tackling these challenges requires an examination of how CALD groups view, participate in and benefit from the participatory research process. It also involves identifying the specific obstacles and enablers that affect their engagement. Such understanding is crucial for developing more inclusive and representative participatory research methodologies [3].

More broadly, there is a general lack of research reporting on experiences of participatory design in suicide prevention [29], despite concerns that such processes can raise problems of burden, safety and power [30]. This is particularly the case with young people, where only a small number of studies have been conducted [4, 6]. The existing evidence suggests that young people find the participatory design in suicide prevention valuable and enjoyable, as it allows them to contribute and make a difference while improving programmes [4, 6]. These studies also highlight the importance of navigating the sensitivities of the topic through wellness plans and environmental design factors that promote safety, inclusivity and comfort. However, such processes have not been assessed specifically with young people from diverse backgrounds such as international students. Thus, there is a need for more research reporting on participatory design and consultation experiences in suicide prevention, particularly with CALD groups.

### 1.1 Current Study

The current study addresses these issues by exploring international students' experiences of co‐consultation to adapt the widely available LivingWorks safeTALK suicide alertness training programme. Two overarching research questions guided the work:

1. What is the perceived value and impact of participating in a suicide prevention co‐consultation process for international students?
2. What are the barriers and enablers that support participation in the co‐consultation process for international students?

## 2 Method

The University of Melbourne Human Research Ethics Committee provided ethical approval for this study (Reference: 27010). The study reports on interview data from international students studying at a University in Melbourne, Australia, who attended a full‐day workshop that commenced with a 3.5‐h standard LivingWorks safeTALK session followed by a 3‐h co‐consultation session to adapt the safeTALK training for their peers. LivingWorks safeTALK is a training programme designed for individuals aged 15+ from the general community that teaches participants how to recognise signs of suicide, start a safe conversation and link individuals experiencing thoughts of suicide with relevant support. The programme includes educational content, example videos and roleplay activities. safeTALK has been shown to be safe and effective in enhancing the knowledge and skills needed to intervene with individuals at risk of suicide among high school students [31], as well as the general population [32], and it is currently part of a large school‐based trial in Australia [33].

### 2.1 Co‐Consultation Session Design

The planning and implementation of the co‐consultation workshop incorporated several considerations. First, all participants completed a wellness plan before the session that included the reporting of any lived or living experience of suicide, other mental health conditions, markers of distress, preferred support methods and safety contacts. The session had four facilitators, including one with experience as an international student, another from a cultural minority group with extensive experience working with international students, another with extensive experience supporting mental health initiatives in education settings and the other a LivingWorks trainer who had delivered the safeTALK training to a diverse range of groups. This approach ensured not only diverse perspectives within the group of facilitators but also meant that there were enough facilitators to help support small group discussions and record ideas and suggestions throughout the co‐consultation process. All safeTALK training sessions are slightly adapted to include relevant support resources for the specific training group. For this workshop, local university counselling services, support contacts and other general helplines for international students were provided.

The workshop environment was intentionally designed to be welcoming, incorporating elements such as low‐volume background music, availability of fidget toys and planned breaks [4]. Additionally, participants were offered the option to take breaks whenever necessary to accommodate their needs. The co‐consultation section of the workshop featured a series of activities, beginning with an icebreaker activity, followed by exercises aimed at enhancing cultural understanding and developing new ideas for the training, before concluding with an in‐depth review of the training content. Key areas for review and potential enhancement included language and key terms, the example scenarios including video vignettes, recommended support services, each of the steps of the intervention model and the roleplay exercises. This structure fostered engagement among participants and facilitators, prompting them to share insights and suggestions for refining the safeTALK programme. Participants also completed a brief post‐session survey where they could provide feedback and request further support if needed. After the session, suggestions for changes were collated and reviewed by the research team and also shared with workshop participants to identify the most important potential changes. Key changes to the safeTALK programme included more detailed definitions of terms such as suicide; an updated list of relevant support services and explanations on how to use interpreters in case of language concerns; roleplay scenarios based on common international student experiences; and more discussion opportunities for students to raise concerns around how they may or may not be able to use the content within their own cultural group. The full workshop schedule and agenda are detailed in Supporting Information S1: Appendix A.

### 2.2 Participants

Participants were eligible to join the co‐design workshop if they were aged 18 years or above and enroled as an international student studying in Victoria, Australia. Recruitment for the co‐design workshop was conducted through an expression of interest form distributed via an email to all international students (> 23,000) at the University of Melbourne and through the local Study Melbourne Facebook page, a service that supports international students in Melbourne, Australia. There were 63 expressions of interest, and purposive sampling was used to select 20 participants for the co‐consultation workshop who were representative of a diverse range of ages, genders, study disciplines and nationalities. We did not ask specifically about lived experience of suicide, as the training is designed for a general audience, although three of the eight interview participants reported lived experiences of suicide in their expression of interest form for the workshop. Seventeen international students attended the co‐consultation workshop, with eight electing to complete an interview about their experience. Interview participants were predominantly females (six females and two males) aged between 22 and 28 years (*M* = 24.5, SD = 2.27) who had lived in Australia for an average of 3.34 years (SD = 2.92, range = 0.5–8 years). Interview participants' home countries included Britain (1), Cambodia (1), China (3), Hong Kong (1), India (1), and Nigeria (1). Participants were enroled in various courses, including biotechnology, construction management, finance, information technology, medicine, nursing, property development, psychiatry and psychology. All participants were reimbursed for participating in the workshop ($150) and interview ($30).

### 2.3 Interviews and Analytic Method

Eight semi‐structured interviews were conducted on Microsoft Teams (Version: 1.6.00.29964). Each interview lasted 15–45 min (*M* = 26.7, SD = 10.9). During the interviews, participants were asked about their experience of the co‐consultation process and whether any elements could be improved for other international students (the complete interview guide can be found in Supporting Information S2: Appendix B). Sessions were audio‐recorded and automatically transcribed by Microsoft Teams. The transcriptions were reviewed for accuracy, and any errors were corrected before analysis.

The research questions for this study aimed to examine students' perception of the value and impact of participating in the co‐consultation along with elements in the process that can be improved. A reflexive thematic analysis of the interview data was conducted using NVIVO 12 [34] software based on the six steps outlined by Braun and Clarke [35]. The analysis was conducted within a paradigmatic framework of interpretivism and constructivism to reflect students' accounts of their attitudes, opinions and experiences more closely while retaining the researcher's reflexive influence on interpretations. This meant we did not use measures of reliability in the analysis, aligning with the tenets of reflexive thematic analysis [35]. Instead, an experiential orientation was taken whereby meaning and meaningfulness of participants were emphasised [35]. The analysis was also primarily inductive, drawing meaning from the data, while deductive structure was based on the research questions.

Interview transcripts were read by CN and re‐read for familiarisation. They were initially read with no specific focus and then coded with a focus on student's experiences with the co‐consultation process. These codes were then reviewed by S.M. and C.N. and sorted into meaningful themes through a process of ongoing discussion and consensus. Themes were then reviewed and refined to ensure a good fit with the data. This process involved searching for data that both confirmed and contradicted the identified themes. In addition to these recognised strategies for ensuring the quality and trustworthiness of qualitative analyses, reflexivity played a central role in the process [36].

### 2.4 Reflexivity Statement

Both authors contributing to the data analysis of this study brought a distinctive perspective shaped by their experiences as international students—one in Australia (5.5 years of study in bachelor, honours and masters programmes) and the other in France (2 years of study in PhD programme). This insider status, as conceptualised by Dwyer and Buckle [37], imbued the analysis with a specific understanding of the international student experience, enabling a deeper connection with the students' perspectives under investigation. The authors also recognised the dual‐edged nature of this insider lens. While it facilitated a richer interpretation of qualitative data by resonating with the lived experiences of the participants, it also posed the challenge of potential bias, where the researchers' experiences might overshadow those of the study participants. To navigate this complexity, the authors actively sought to consider how their perspectives may be influencing the interpretations of the data during data collection and analysis. This included reflecting to participants the interviewers' understanding of what was described during interviews, the inclusion of multiple members of the team in analysing and reviewing the results and integrating the findings into the larger literature.

## 3 Results

### 3.1 Research Question 1: What Is the Perceived Value and Impact of Participating in a Suicide Prevention Co‐Consultation Process for International Students?

In terms of what students found valuable and impactful in participating in the co‐consultation process, we identified four broad themes termed empowerment and recognition, shared learning, enjoyable experience, and drawing on diverse experiences to develop solutions. Each of these are outlined in detail below and illustrative quotes are included in Table 1.

**Table 1:** Illustrative data for themes.

Theme	Illustrative quotes
Empowerment and recognition	1. Actually, I think I should be the person who says thank you because it's like targeting international students' mental well‐being. Yeah, like, I really appreciate the programme and all the efforts you guys make for international students. That's quite touching, actually (participant 1) 2. It's a very tricky group cause people think international students are rich. They are here; how could they have mental health problems? How could they have challenges? But in reality, there are lots of problems out there. So, I'm really touched. There are really a group of researchers trying to help people, especially international students, to help tackle these kinds of problems or challenges (participant 2) 3. As an international student, we can talk about our own questions, and also, from our experience, we know that we can help others. So, I think that's really, it's a really valuable part (participant 3) 4. A lot of people attending were very open about, well, if I was back home, this would be happening, or if I was speaking with my family, this would happen, so I don't think (there was anything missing) because people brought that experience with them. So, I think it felt like it was a good way of doing it because it wasn't assuming who would be there and what their experiences would be. It was allowing people to bring that to the discussion (participant 6)
Shared learning	5. … He also mentioned the point that talking about suicide is still difficult [in India]. Yeah, and that kind of like I'm thinking in a similar way. Yeah, and I realised that the cultural complexity is really a thing … The suicide topic is more complex than I imagine because for the [safeTALK] workshop part, everything looks so smooth, but for the codesign part, everyone is giving critical ideas (participant 1) 6. Hearing others, how different cultures shape people's perception about suicide, it's actually an opportunity for me to learn new knowledge and know different culture norms (participant 2) 7. I was able to also hear from other people and most of them, I think were from South East Asia. They had similar experiences with where I was coming from. So, I really enjoyed that part (participant 4) 8. That was the first thing I wanted to do, to learn, to know more about it, to kind of match it with my own experience, to see, you know, something that I've experienced maybe is actually what I thought it was. And then I was able to meet other people and also learn from other cultures. That is how they see it in their own cultures and your religion. Yeah, so it was really nice (participant 4)
Enjoyable experience	9. Yes, I think it met my expectations, especially in the afternoon time, I did not feel sleepy at all. I was really excited, I really liked that we had a small group table discussion and I remember you were in my group to guide the discussion, and everyone had that opinion to share, but of course, it will be a few times like everyone just stopped to keep silent, but you will throw a question for us to keep thinking. I really like that (participant 2) 10. I really like how he (the facilitator) started by asking us. OK, so a gift. Like, what do you think? I really like that part, actually (referring to the ice‐breaker task). So, I think for me, the experience is very good. It's very engaging and I actually shared a lot of thoughts … I'm really happy to see there are so many international students willing to contribute their thoughts and ideas to this workshop. (participant 2) 11. Kind of individual time so you are able to speak about what you think and how you feel without being confronted by having to present to everyone. I thought that was a really nice way of doing it (participant 6) 12. I was talking with my like Chinese fellows. We actually talked a lot … It's like a deeper conversation. (participant 1) 13. … I think even the students around me and the peers that we were talking on the table, everyone was pretty comfortable discussing everything because I think we all knew that this is for the good and that's why we are developing it and like that's why the workshop is important, all the content might be distressing. I didn't feel it (participant 5) 14. We all joined the workshop with the same purpose of contributing to the international student perspective. So, I think the co‐design in the afternoon is really good (participant 8) 15. I did not feel burdened by the topic, to be honest, and another fun part about it was us being able to work together. So that really made the atmosphere more light and helped us speak through it in a more comfortable way than I would have imagined it to be if it was not like in that kind of space and more of a one‐on‐one space that might have been more challenging to think about more ideas and get that peripheral view on everything (participant 5)
Drawing on diverse experiences to develop solutions	16. I think that's really interesting to work [with] people from different backgrounds. Yeah, I think it's a good way because it can gather lots of international students together and it can generate lots of ideas from these people (participant 3)
Barriers and enablers of participation for international students	17. I think no matter what we are saying … the people in the room will not judge me and we also have some like some toys to help us to relax … So I think that's good. (participant 3) 18. The workshop is, it's creating a very safe environment. Besides, we don't actually know each other, so the stuff that we talk about … the privacy is there, I would say. (participant 8) 19. With the codesign session, I realised that we were split off into our groups, but also brought back together. So before the next question went on, having that kind of individual time so you are able to speak about what you think and how you feel without being confronted by having to present to everyone. I thought that was a really nice way of doing it. (participant 6) 20. I can't think of any other way that it could like it could be facilitated otherwise. But this workshop was very well organised like having the session first and then trying to make it better. So that was amazing. (participant 5) 21. … I remember we have a couple of tables and at my table like half of the members (3 out of 6) are from China. So, I guess somehow I'm really interested to hear other students perspectives, so if there is an opportunity for each table to share … I was just really curious about other people's thoughts as well (participant 2) 22. It might be potentially useful to hear from individuals, so maybe have like a form where individuals can say exactly their points, because obviously again there might be people who aren't that comfortable discussing in a group, but they might have quite passionate ideas about what could have been changed or what would have been better (participant 6) 23. We talked in English, so that was good … [but] in a lot of other languages, the word suicide, mental, and things around that are used very, very differently or they don't even exist. And this was mentioned in the workshop and the co‐consultation process. But I think that would be something to consider when facilitating the workshop. The language is something that I would really be focused on if I were to do it again (participant 5)

#### 3.1.1 Theme 1: Empowerment and Recognition

International students who participated in the suicide prevention co‐consultation process reported a sense of empowerment and recognition. This was identified in two forms. First, students felt valued by being the focus of the research and intervention. Second, participants felt empowered by having a voice in shaping a suicide prevention programme for other international students.

Participants appreciated that the research and intervention were centred around addressing the mental well‐being of international students (see Table 1, quote 1). Students felt the co‐production process also counteracted common stereotypes regarding international students and mental health by recognising their diverse experiences and challenges (see Table 1, quote 2). The results also highlighted that international students may feel overlooked or marginalised by current systems and supports and simply focussing on them makes them feel valued.

The session also gave students a platform to share their personal experiences, provide feedback and contribute their opinions on the critical topic of suicide prevention for international students. They reported that having an opportunity to improve support for other international students was perceived as valuable and worthwhile (see Table 1, quote 3). Through the session, participants were also able to identify how their cultures and different cultural contexts might influence their use of the training content (see Table 1, quote 4). Students were willing to share their experiences and felt empowered by the opportunity to share their perspectives and ideas. Similarities and differences across the different cultures represented in the room were also identified through this process. This helped to facilitate shared learning and opportunities to enhance the training relevant to multiple international student groups.

#### 3.1.2 Theme 2: Shared Learning

Participants recognised the significance of learning as a key aspect of their co‐consultation experience. Students appreciated the opportunity to learn from each other and critically reflect on the training content. One notable insight was the recognition that suicide is a complex issue, particularly within diverse cultural communities, contrary to the simplified portrayal in the training. Increased awareness of complexity was important but also led participants to question the sometimes simplified views portrayed in the training (see Table 1, quote 5). The learning process also exposed participants to other cultural perspectives and ideas on suicide (see Table 1, quote 6). At the same time, there were also shared perspectives found between the cultural groups (see Table 1, quote 7). The reassurance of being able to relate to the mental health and cultural experiences of others further enhanced the learning experience (see Table 1, quote 8). Throughout this process, students felt it was very important to be open‐minded. They also highlighted the importance of cultural awareness and empathy to support the shared learning process.

#### 3.1.3 Theme 3: Enjoyable Experience

Contrary to the participants' expectations, they consistently described the co‐consultation process as a fun, engaging and enjoyable experience. This was, in part, attributed to the way the session was structured and facilitated. Participants reported that facilitators helped ensure all voices were heard, guided discussions and captured significant insights and ideas generated during the co‐consultation (see Table 1, quote 9). Similarly, environmental factors played a key role in supporting participant well‐being. Including stress‐relieving toys (e.g., fidget toys) and early warm‐up activities created a safe environment for participants with their well‐being in mind and acted as icebreakers that helped make the session more engaging (see Table 1, quote 10). Participants also said they appreciated the small group dynamics, where they had the time to connect with others without having to share within a large group (see Table 1, quote 11). These small groups sometimes facilitated more in‐depth discussions, particularly among people with shared experiences or cultural backgrounds (see Table 1, quote 12). Similarly, despite the sensitivity of discussing suicide, participants highlighted the workshop's structured approach and safe environment meant it was not distressing (see Table 1, quote 13). Lastly, participants found working together enjoyable as it felt like they were contributing to a good cause (see Table 1, quotes 14 and 15).

#### 3.1.4 Theme 4: Drawing on Diverse Experiences to Develop Solutions

Diversity emerged as a crucial factor contributing to solutions in the co‐consultation process. Recognising co‐consultation as a creative process, participants valued the openness of fellow students in sharing their experiences and culture. This contributed to creative solutions and a more comprehensive discussion (see Table 1, quote 16). For example, the peer trainer model was suggested by students as a way to adapt the training to make it more culturally relevant. An international student or someone who would be able to relate to the international student experience would be trained to deliver the training and guide discussions.

### 3.2 Research Question 2: What Are the Barriers and Enablers That Support Participation in the Co‐Consultation Process for International Students?

A small amount of feedback was provided regarding the barriers and enablers that supported or hindered participation in the co‐consultation process. This limited feedback did not lend itself to the development of overarching themes but instead was better captured as single suggestions. In terms of enablers, the session design features, including ice‐breaker activities, ground rules, ongoing emphasis on self‐care and wellness plans, were all identified as factors that helped participants feel safe to participate (see Table 1, quotes 10, 17 and 18). Regular breaks that included catered food and drinks also helped participants connect in a less formal manner that supported later engagement. A mix of activities and small and large group exercises supported participants to develop ideas while also hearing from the larger group (see Table 1, quote 19). Starting with the safeTALK training followed by the adaptation phase also supported students to have a more of a shared understanding of the topic that they could use when discussing potential programme enhancements (see Table 1, quote 20).

Key barriers to participation related to limited opportunities to interact with other participants, a focus on group sharing that was not comfortable for all participants, and issues around shared language. Participants desired more opportunities to interact with a wider range of perspectives and opinions. Although organising group membership based on cultural similarities was seen as valuable for in‐depth discussions, there was a desire for rotating group membership to facilitate greater interaction across participants (see Table 1, quote 21). Participants also highlighted the importance of creating spaces for quieter voices to be heard and understood. To address this, they suggested offering additional feedback channels, such as surveys and interviews after the workshop. These channels could encourage the people who are quieter and may be less comfortable speaking in a group setting to provide their feedback and concerns (see Table 1, quote 22). Some participants suggested that online environments may be helpful for addressing these issues and improving accessibility of the co‐consultation process. Lastly, it was acknowledged that the language surrounding suicide and mental health varies across cultures and that these needed to be addressed to ensure effective communication during co‐consultation (see Table 1, quote 23).

## 4 Discussion

This study explored international students' experiences of participating in a suicide prevention co‐consultation process to adapt the LivingWorks safeTALK suicide alertness training programme. Participants characterised the co‐consultation experience as empowering and enjoyable, highlighting that it fostered learning and effectively leveraged their diverse perspectives. Students felt valued by being a focus of the research and appreciated having a voice in shaping the suicide prevention programme. The process encouraged shared learning among participants from different backgrounds, challenging simplified views of suicide and prompting greater cultural awareness. Despite the serious subject matter, students described the session as engaging and not distressing due to skilled facilitation and a well‐structured environment. Finally, participants valued opportunities to share diverse experiences, leading to a variety of potential adaptations for the suicide prevention programme. Suggestions for improving the co‐consultation process included providing more opportunities for interaction among participants, incorporating alternative communication channels for quieter voices and addressing language differences to improve the inclusivity and effectiveness of the co‐consultation process.

Our results support and expand upon the broader participatory design literature with youth and CALD communities by showing that such methods can empower international students while supporting the cultural adaptation of suicide prevention programmes [3]. There is a growing body of evidence that participatory design in suicide prevention with youth populations is both valuable for participants and prevention initiatives [4, 6, 27]. Specifically, young people feel empowered by contributing to programme development while simultaneously enhancing the programmes' ability to meet their needs [28, 38]. The findings suggest that similar outcomes occur for international students.

A key contributor to the success of participatory design in youth suicide prevention is the underlying planning, logistics and safety considerations. Studies have shown that it is essential to create safe, inviting environments that meet participants' needs [4, 6]. We implemented these approaches by integrating wellness plans, ground rules, facilitators with various lived experiences and environmental factors such as music and fidget toys (see co‐consultation session design section). These features helped to create a supportive environment where students could participate safely and meaningfully in the design process. They may have also contributed to the lack of power imbalance issues that were reported in our study, which can be an issue during participatory design [30].

Similarly, despite concerns that participatory design in suicide prevention could be distressing for some individuals [4], none of our participants reported feeling distressed during the training or co‐consultation phases. Prior evidence with young people showed that co‐designing suicide prevention content could make a small number of people (6.1%) with lived experience of suicide feel suicidal [4]. In our study, we did not specifically seek individuals with lived or living experiences of suicide, as we were primarily focused on international student experiences. Despite this, a few participants noted lived experiences of suicidality and other mental health issues but also did not report any issues during the co‐consultation workshop. The lack of distress among participants might also be attributed to our training and adaptation focussing on methods to adjust suicide alertness training rather than directly discussing methods to communicate about experiences with suicide online, as was done in the previous #chatsafe research [4]. This is a plausible explanation, as the other study exploring co‐design experiences of young people to design a post‐suicide support service for young people also had no reports of distress through the process [6].

A particularly pertinent finding in our study underscores the capacity of shared experiences among international students to serve as a foundational basis for culturally adapted suicide prevention strategies within this diverse group. One of the significant challenges of suicide prevention and other mental health interventions with international students is the extensive cultural diversity within international student cohorts [2]. This diversity is considered a significant barrier because it can be challenging to provide programmes that effectively respond to different cultural perspectives or needs [2]. While this is a valid concern and should be a key consideration in all work with international students, our participants reported that the diversity of participants acted as a strength to promote varied perspectives and ideas while highlighting the shared experiences and culturally specific experiences between groups. For instance, aligning with a variety of existing literature [25, 39, 40], many students discussed similar experiences with cultural stigma related to mental health, generational gaps in mental health literacy and difficulties with negotiating this landscape. Similarly, students described shared challenges related to being an international student, such as adapting to the local host culture and education system [41]. These shared bridging experiences were viewed as key areas for adaptations that could be relevant to various international student groups, while specific needs could also be addressed through alternative pathways such as specific language adaptations or other tools. This finding suggests that the shared experiences of international students may provide an effective foundation for universal prevention programmes applicable to diverse international student groups.

Our participants highlighted opportunities to better support international students and other migrant groups in engaging in participatory design. Central to these were ways to improve communication and engagement between participants and researchers. Whether the focus was on increasing engagement between participant groups, supporting those with quieter voices to be able to provide feedback or addressing cultural or language misunderstandings regarding topics such as suicide, the suggestions all centred on breaking down potential barriers to communication, sharing and learning. Enhanced participant engagement and the incorporation of language and cultural insights are in line with recent guidance suggesting that co‐design with CALD communities should foster the development of a shared explanatory model between researchers and participants that mirrors the community's experiences and perspectives [3, 42]. This is particularly pertinent for international students who bring diverse cultural perspectives on mental health that may not align with the Western biopsychosocial models of mental health [43]. Sharing these different views during participatory design may facilitate learning for both participants and researchers while improving our understanding of effective methods to communicate about suicide with international students.

### 4.1 Limitations

There are several limitations to consider in our study. First, we assessed participants' experiences adapting an existing widely used suicide alertness prevention programme through co‐consultation. Studies involving more extensive co‐design that draw directly upon lived experience of suicide may have greater iatrogenic effects. Similarly, we only had a small number of participants representing a limited number of nationalities and international student experiences. Other international student groups might navigate the co‐design process differently, potentially encountering power imbalances or culturally specific challenges. Future research should continue to report on youth participant experiences of participatory research in suicide prevention and mental health more broadly to provide a broader understanding of how, when and why participatory research is beneficial or challenging.

## 5 Conclusion

International students are a key group at risk of suicide who rarely engage with the relevant supports or services. Participatory design can be used to adapt suicide prevention training or other mental health literacy programmes that can encourage help‐seeking. Similar approaches may also support the co‐designing of new international student‐specific programmes that are designed by and for international students, although further research is required to test this contention. Our study shows that co‐consultation with international students appears to be safe, empowering and valued by participants while also supporting researchers to adapt existing suicide prevention programmes to better meet international students' needs. Specifically, the views from participants will inform the adaptation of the safeTALK training for international students so that it is better aligned with their cultural needs and experiences in the future.

## Author Contributions

**Samuel McKay:** conceptualization, methodology, software, data curation, investigation, formal analysis, supervision, funding acquisition, project administration, resources, writing–original draft, writing–review and editing. **Christina Ng:** data curation, investigation, formal analysis, writing–original draft, writing–review and editing, project administration. **Bridget Kenny:** methodology, project administration, writing–review and editing. **Rafi Armanto:** methodology, writing–review and editing. **Michelle Lamblin:** methodology, project administration, writing–review and editing. **Jo Robinson:** resources, supervision, writing–review and editing.

## Conflicts of Interest

The authors declare no conflicts of interest.

## Supporting information

Supporting information.

Supporting information.

## Data Availability

Research data are not shared due to the ethical constraints of the study.
