# Supplementary material for: Participatory Design in Suicide Prevention: A Qualitative Study of International Students' Experiences of Adapting the LivingWorks safeTALK Programme
Source: Health Expect. 2024 Aug 6;27(4):e14164. doi: 10.1111/hex.14164 (PMC11303664; doi:10.1111/hex.14164)
Supplement: Supplementary file 1 — Supporting information. [file HEX-27-e14164-s002.docx]

**Schedule**

9:00am-9.15am - Intro and Welcomes

9:15am-11:00am – safeTALK

11:00am-11.15am - Break

11:15am-12:45pm - safeTALK training

12:45pm-1:15pm Lunch

1:15pm-2:45pm - safeTALK adaptation pt1

2:45-3:00pm - Break

3:00-4:30pm - safeTALK adaptation pt2

**Agenda Intro and welcomes (15 minutes)**

- Welcome and thank participants for attending.
- Introduce the team running the workshop.
- Agenda and aims for the day.
- Key considerations for the day:
  - Confidentiality.
  - Respect and listening.
  - Inclusive language.
  - Safe space commitment.
  - Support availability.
  - Expertise across all groups.
  - Emphasise questions and feedback welcome.
- Brief introductions from participants.

**safeTALK adaptation pt1 (1.5 hours)**

- **3 minutes (1:15): Introduction to co-design/adaptation process**
- **2 minutes:** **Reminder of ground rules for the workshop**
- **20 minutes (1:20): Cultural understanding exercise -** Begin with an exercise that aims to enhance the understanding of different cultures present among the participants.
  - What are the common beliefs about mental health, suicide, or suicide prevention in your culture? (Explore differences by age, gender, and subgroups (e.g., religious groups, etc.) within each culture).
  - Are there other cultural norms in your culture that you think we should consider during this workshop or the adapted SafeTALK workshop?
  - How would you suggest we integrate or address these beliefs in the workshop, if at all?
  - Could we use these ideas to increase participant engagement in the workshop?
- **20 Minutes (1:40): General idea generation**: Encourage participants to generate ideas on how to adapt the safeTALK training to better meet the needs of international students. This could include suggestions for making the program more culturally inclusive, adding sections addressing unique stressors, adapting exercises, or suggestions for language accessibility. Split into five groups of four and give them 10 minutes to work on ideas independently before bringing students back into the larger group
- **15 minutes (2:00): Scenario generation exercise** — Go through the scenarios shown in the training videos (e.g. school lockers, the phone call from mum, basketball courts, counselling session, etc.). What are some common situations where international students may discuss thoughts of suicide or share invitations? More broadly, what are the common situations where international students interact with others?
- **15 minutes (2:15): Support services** — who would you contact for support? What professional services would you view as acceptable? What barriers to accessing these services should be addressed in the training?
- **15 minutes (2:30): User persona development including language adaptation** — Use this tool to create detailed user personas representing different types of international students. Split students into groups and ask them to generate three personas each that include factors such as their country of origin, language proficiency, social situation, reasons for studying abroad, etc. Bring the group back together and ask them to share the personas. Based on what is shared, ask the group if they think it will be possible to have a single international student-specific SafeTALK training program based on the shared international student experience or if there is too much cultural diversity?

**Break (2:45 - 3:00)**

**SafeTALK adaptation pt2 (1.5 hours)**

Reviewing the key steps of the TALK model

- **15 minutes (3:00): TALK Step Review**: Review each step of the TALK model, and discuss how cultural factors and the experiences of international students might affect each step. For instance, in some cultures, it might be less acceptable to directly 'Ask' someone about suicidal thoughts, so participants might brainstorm alternative approaches.
- **15 minutes (3:15) Adaptation Brainstorming**: Ask participants to brainstorm ways to adapt each TALK step to be more effective and sensitive to international students' experiences and cultural backgrounds. Encourage creativity and inclusivity.
- **20 minutes (3:30): Prototyping**: Have participants sketch or write out their adapted TALK steps. They could also develop example scenarios to demonstrate how these adaptations would work in practice.
- **20 minutes (3:50) Role-Playing and Feedback**: Run a few role-playing scenarios using the adapted TALK steps. After each scenario, gather feedback from observers and the role-players themselves. How did it feel? What worked well? What might need to be changed?
- **10 minutes (4:00): Refinement**: Use the feedback from the role-playing exercise to refine the adapted TALK steps further. This may involve several rounds of feedback and refinement to ensure effective adaptations.
- **10 minutes (4:10): Reflection and Implementation Plan**: At the end of the workshop, reflect on the process and discuss how these adapted TALK steps and other feedback can be implemented. Ask for any suggestions on topics or ideas that were not discussed during the workshop.
- **10 minutes (4:20) 2-item Survey + general comment feedback 5 minutes + interview invitation**
