# Supplementary material for: Participatory Design in Suicide Prevention: A Qualitative Study of International Students' Experiences of Adapting the LivingWorks safeTALK Programme
Source: Health Expect. 2024 Aug 6;27(4):e14164. doi: 10.1111/hex.14164 (PMC11303664; doi:10.1111/hex.14164)
Supplement: Supplementary file 2 — Supporting information. [file HEX-27-e14164-s001.docx]

***Introduction***

General introduction to interview: My name is [interviewer name] and I am a researcher from [university name]. For this research we are evaluating students’ experiences of the co-consultation workshops to adapt the SafeTALK training for international students. During this interview, I will ask you questions about your experience. It’s important to note that there is no right or wrong answer to my questions. What will be the most helpful for me is if you give responses with as much detail as you feel comfortable sharing with me. I will guide you through the interview so that we finish it all in an hour or less and I will take handwritten notes to remind me of important ideas. Do you have any questions before we get started?

***Questions***

1. What motivated you to join the workshop?
   1. Have you participated in a similar workshop before?
2. Did the workshop meet your expectations?
3. What did you enjoy most about the workshop?
4. What did you enjoy least about the workshop?
5. Did you feel that your voice and perspective were heard and valued during the co-consultation workshop?
6. Were there any aspects of the workshop that you found confusing or challenging?
7. Did you learn new things during the workshop?
8. Did you develop any new skills during the workshop?
9. Have you used knowledge or skills that you just mentioned since completing the workshop?
   1. How so/why not?
10. How could the co-consultation process or workshops be improved for international students in the future?
    1. Was the format appropriate (e.g., initial training, opportunity for review and development)?
    2. Did you feel comfortable during the workshop?
    3. Was any of the content distressing? Are there ways it could be made less distressing?
11. Were there any cultural considerations or perspectives that you felt were missing or could have been better represented during the co-consultation workshop?
12. Were there any outcomes or results from the co-consultation workshop that you are particularly excited about or interested in exploring further?
13. Overall, do you feel that the co-consultation workshop was a valuable and effective approach to adapting suicide prevention training for your community? Why or why not?
14. Is there anything else you think I should know before finishing up the interview?
